# Genotyping of ticks: first molecular report of *Hyalomma asiaticum* and molecular detection of tick-borne bacteria in ticks and blood from Khyber Pakhtunkhwa, Pakistan

**DOI:** 10.3389/fcimb.2024.1346595

**Published:** 2024-03-12

**Authors:** Muhammad Kashif Obaid, Shehla Shehla, Guiquan Guan, Muhammad Rashid, Sumaira Shams

**Affiliations:** ^1^ State Key Laboratory for Animal Disease Control and Prevention, Key Laboratory of Veterinary Parasitology of Gansu Province, Lanzhou Veterinary Research Institute, Chinese Academy of Agricultural Sciences, Lanzhou, Gansu, China; ^2^ Department of Zoology, Abdul Wali Khan University Mardan, Khyber Pakhtunkhwa, Pakistan; ^3^ Department of Parasitology, Faculty of Veterinary and Animal Sciences, The Islamia University of Bahawalpur, Bahawalpur, Punjab, Pakistan

**Keywords:** *Anaplasma phagocytophilum*, *Hyalomma asiaticum*, *Candidatus* Rickettsia shennongii, *Ehrlichia*, blood-borne pathogens

## Abstract

Multiple ticks (Acari: Ixodoidea) carrying Rickettsiales bacteria have significant importance for both human and animal health. Thus, the purpose of this work was to genetically analyze tick species and their associated Rickettsiales bacteria in animal hosts. In order to achieve these objectives, various animals (including camels, cattle, goats, sheep, dogs, and mice) were inspected in four districts (Mardan, Peshawar, Kohat, and Karak) of Khyber Pakhtunkhwa to collect ticks, while blood samples were collected from all the symptomatic and asymptomatic cattle in all four districts. A total of 234 ticks were obtained from 86 out of 143 (60.14%) host animals, which were morphologically identified as *Rhipicephalus turanicus*, *Rhipicephalus microplus*, *Haemaphysalis cornupunctata*, and *Hyalomma asiaticum*. Among these, their representative ticks (126/234, 53.85%) were processed for molecular confirmation using cytochrome *c* oxidase (*cox1*) gene. Obtained *cox1* sequences of four different tick species showed 99.72%–100% maximum identity with their corresponding species reported from Pakistan, China, India, and Kazakhstan and clustered phylogenetically. This study presented the first genetic report of *Hy. asiaticum* ticks in Pakistan. Moreover, genetically confirmed tick species were molecularly analyzed by PCR for detection of Rickettsiales DNA using partial fragments of *16S rDNA*, 190-kDa outer membrane protein A (*ompA*), and 120-kDa outer membrane protein B (*ompB*) genes. In addition, blood samples were analyzed to identify Rickettsiales bacteria using the aforementioned genes. Rickettsiales bacteria were found in 24/126 (19.05%) ticks and 4/16 (25.00%) in symptomatic cattle’s blood. The obtained *ompA* and *ompB* sequences from *Hy. asiaticum* ticks showed 99.73%–99.87% with *Candidatus Rickettsia* shennongii and unidentified *Rickettsia* sp., whereas the obtained *16S rDNA* sequences from cattle’s blood and ticks (*Hae. cornupunctata*) showed 99.67% highest identity with *Anaplasma phagocytophilum*. The *16S rDNA* sequence of Rickettsiales DNA from *Rh. turanicus* ticks showed 100% identity with *Ehrlichia canis* and unidentified *Ehrlichia* sp. Obtained sequences of Rickettsiales bacteria were grouped along with their respective species in phylogenetic trees, which were previously reported in Greece, Cuba, Iraq, Turkey, Pakistan, South Korea, and China (mainland and Taiwan). This extensive study explores the wide range of damaging ticks and their corresponding tick-borne bacteria in the area, suggesting a possible danger to both livestock and human communities.

## Graphical abstract

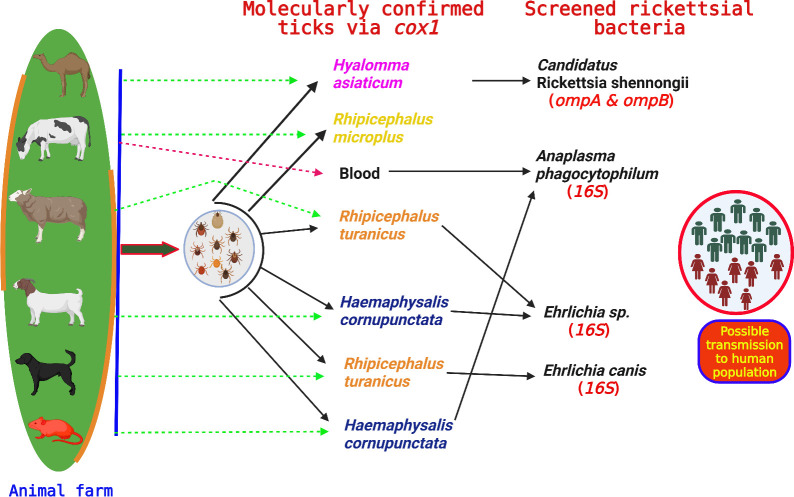

## Introduction

Ticks (Acari: Ixodoidea) play a significant role in the maintenance and spread of numerous zoonotic protozoans, bacteria, and tick-borne viruses, posing a significant risk to the health of people and animals worldwide (Bekloo et al., 2018). The infections caused by these tick-borne pathogens (TBPs) lead to significant losses in livestock production in tropical and subtropical regions. This has substantial economic consequences since it reduces farmers’ revenue and poses a challenge to the cattle industry in global markets (Jongejan and Uilenberg, 2004). In addition, the presence of theileriosis has a significant effect on livestock farms in Pakistan, leading to a decrease in milk output and an increase in animal deaths (Rashid et al., 2018).

Several prominent tick species from different genera, such as *Rhipicephalus*, *Hyalomma*, *Haemaphysalis*, *Ixodes*, *Argas*, *Ornithodoros*, *Nosomma*, and *Carios* have been found in diverse locations in Pakistan, infesting both humans and animals (Karim et al., 2017; Ali et al., 2019; Aiman et al., 2022; Numan et al., 2022; Zahid et al., 2023). Various tick species are responsible for the transmission of numerous bacterial pathogens including *Rickettsia* spp., *Anaplasma* spp., *Ehrlichia* spp., and *Coxiella* spp. to different host animals like cattle, buffaloes, dogs, camels, mice, goats, sheep, domestic fowls, and equids (Khan et al., 2022; Ali et al., 2023a; Ali et al., 2023b; Shehla et al., 2023a). In addition, many TBPs such as *Babesia* spp., *Theileria* spp., *Hepatozoon* spp., *Rickettsia* spp., *Anaplasma* spp., and *Ehrlichia* spp. have been identified in blood samples taken from various domestic animals, including goats, sheep, dogs, cattle, and buffaloes (Ahmad et al., 2018; Hassan et al., 2018; Malik et al., 2018; Iqbal et al., 2019; Khan et al., 2020; Iatta et al., 2021).

The order Rickettsiales comprises the genera *Rickettsia*, *Anaplasma*, and *Ehrlichia*, which are obligate intracellular gram-negative bacteria that replicate inside the host's cells of host's body (Schmutzhard and Helbok, 2014). *Rickettsia* possesses a diverse spectrum of arthropod vectors and vertebrate hosts for their transmission. The scope and influence of the recognized tick-borne Rickettsiales pathogens have significantly expanded in the last 25 years, making this intricate category of diseases an ideal model for studying emerging and reemerging infections. Many tick-borne rickettsiae that were formerly thought to be harmless are now believed to be associated with human illnesses. Additionally, new species of *Rickettsia*, with unknown levels of virulence, are constantly being discovered in ticks worldwide (Parola et al., 2013). Based on the biological and genetic characteristics, rickettsiae are classified into four groups: the Spotted Fever Group (SFG), Typhus Group (TG), Transitional Group (TRG), and Ancestral Group (AG) (Gillespie et al., 2007). Tick-borne Spotted Fever Group Rickettsiae (SFGR) are globally distributed and can cause life-threatening illnesses, such as Rocky Mountain spotted fever and Mediterranean spotted fever (Paddock and Alvarez-Hernández, 2018). In Pakistan, different *Rickettsia* species belonging to the SFG including *Rickettsia massiliae*, *Rickettsia hoogstraalii*, *Candidatus Rickettsia* shennongii, and *Rickettsia* spp. have been molecularly documented in various tick species (Ali et al., 2023b; Shehla et al., 2023a).

In addition, many species of *Anaplasma* are accountable for inducing anaplasmosis in both people and animals, which is a tick-borne disease (TBD) and characterized by the destruction of red blood cells (Kocan et al., 2003). The illness is becoming an escalating and significant menace to the animal breeding system. It causes an extra burden on animal healthcare due to its infection, which results in decreased body weight, reduced milk supply, and frequent abortions in animals, ultimately leading to death (Stuen et al., 2002; Stuen et al., 2003). *Anaplasma* species, such as *Anaplasma phagocytophilum*, *Anaplasma centrale*, *Anaplasma capra*, *Anaplasma platys*, and *Anaplasma marginale* have been genetically analyzed in numerous arthropod vectors and host animals worldwide (Byaruhanga et al., 2018; Iqbal et al., 2019; Ali et al., 2023b). Specifically, the *A. phagocytophilum* is regarded as an important zoonotic pathogen, causing disease in humans, domestic ruminants, horses, dogs, and rarely in cats, while wild mammals act as its reservoir host (Dugat et al., 2015). This obligate intracellular Rickettsiales pathogen replicates in neutrophilic granulocytes and leads to thrombocytopenia, leukopenia, anemia, and immunosuppression associated with variable clinical signs (Dumler et al., 2005). Similarly, the genus *Ehrlichia* is also another highlighted TBP, and its different species have been molecularly documented in tick species, humans, domestic animals (cattle, buffaloes, horses, goats, sheep, and dogs), and wild animals including coyote, white-footed mice, and white-tailed deer (Dumler et al., 2001; Ali et al., 2023b).

Main TBDs, often referred to as blood-borne diseases (BBDs) such as anaplasmosis, babesiosis, theileriosis, and ehrlichiosis have a significant impact on bovines in Pakistan, particularly in terms of economic losses (Jabbar et al., 2015; Malik et al., 2018). Livestock plays a crucial role in the animal production sector, making a significant contribution of approximately 11.8% to Pakistan’s yearly gross domestic product (GDP). The agricultural sector is primarily run by small-scale farmers who cultivate essential nutrients and proteins to provide food security and generate revenue for the nation (Jabbar et al., 2015). Nevertheless, the growing number of TBDs has a negative impact on the agricultural and animal industries in Pakistan. Hence, this study was aimed for molecular identification of various tick species and their associated Rickettsiales pathogens in Khyber Pakhtunkhwa (KPK), Pakistan.

## Materials and methods

### Study area description

**Figure 1:**
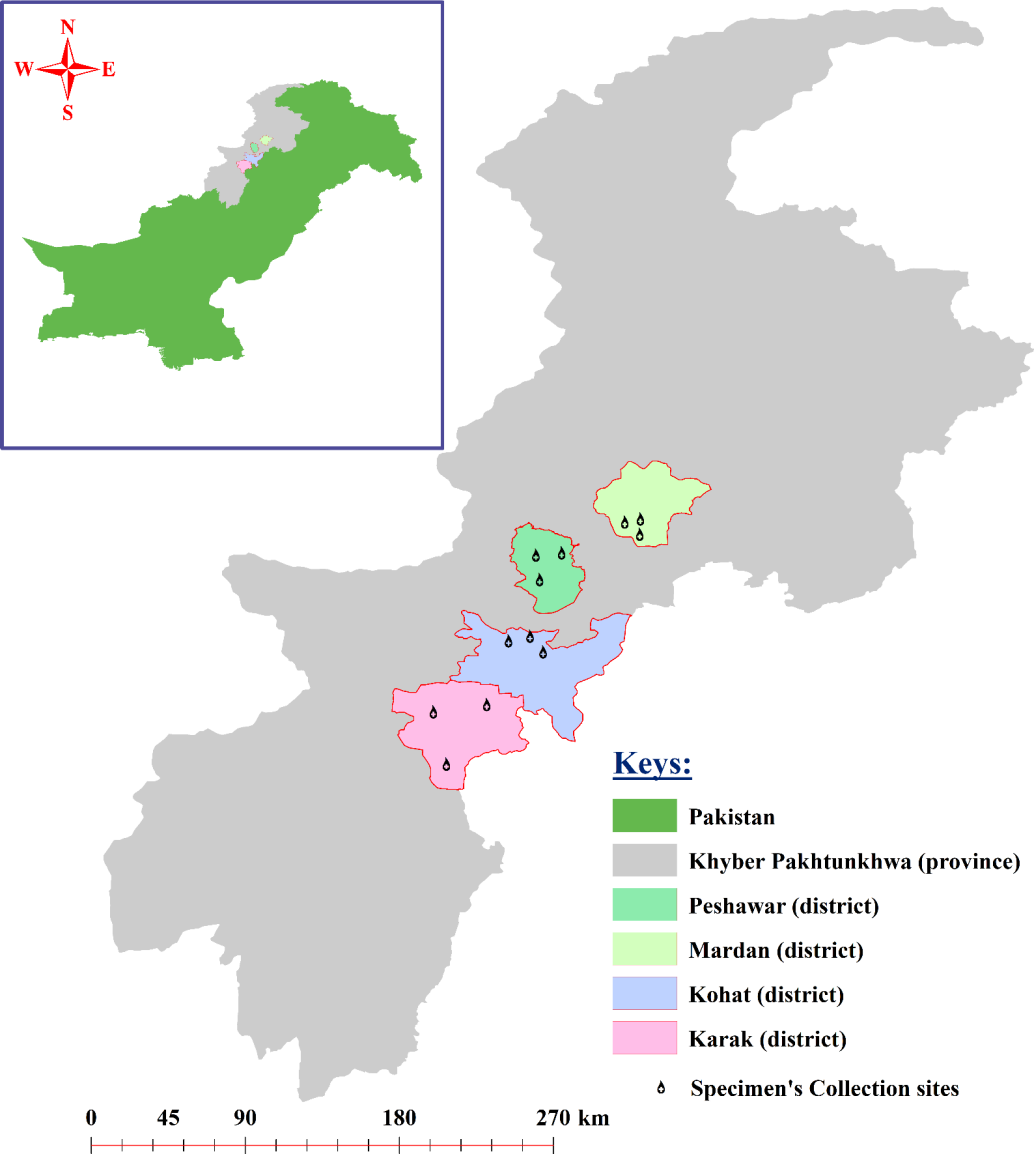
Study map of the four districts of the study area where the specimen collections were performed.

Khyber Pakhtunkhwa is one of the colder province in Pakistan and various districts including Mardan (34.1986°N, 72.0404°E), Peshawar (34.0398°N, 71.5668°E), Kohat (33.5828°N, 71.4627°E), and Karak (33.1335°N, 71.0365°E) of KPK were selected to cover the current study. These districts are comprised of both lower and upper highlands as well as plain areas. The study region exhibits a climate that ranges from dry to semi-arid, with an average annual temperature fluctuating between 8.5°C and 33.4°C. The average humidity levels vary from 28% to 82% throughout the duration of the year. Likewise, the annual precipitation in the given regions varies from 15 mm to 200 mm (https://en.climate-data.org/asia/pakistan/khyber-pakhtunkhwa-2239; accessed on October 16, 2023). Different factors like temperature, humidity, and precipitation have major influence on the growth and distribution of various ticks (Karim et al., 2017). The geographical coordinates of the study locations were obtained using global positioning system (GPS) and inserted into Microsoft Excel 2003 (Microsoft 365®) to design a study map using the ArcGIS V. 10.3.1 (ESRI, Redlands, CA, USA) (**Figure 1**). Three sampling points/locations in each district are represented by a drop-like structure in **Figure 1**.

### Collection and morphological identification of tick specimens

From March to August 2023, a total of 12 animal farms (three from each district) in the aforementioned districts were surveyed to collect ticks from different hosts such as camels, cattle, goats, sheep, and dogs. In addition, a substantial number of live mice were seized irregularly, to collect ticks from them, using plastic snap traps, which were installed/placed at different places in the studied livestock farms. Ticks were collected from various body parts like the sternum, belly, anal region, udder, legs, head, and neck region, using forceps that were applied as close as possible to the attachment point on the host’s body to pull the tick. Verbal and written permission (consent) was also taken from the animal farm’s owners before the sample collection. Ticks were collected once from each infested animal host in each farm in various districts. Ticks collected from each specific host in each district were placed separately in a labeled Eppendorf tube and were brought to the Laboratory of Molecular Parasitology, Department of Parasitology, Faculty of Veterinary and Animal Sciences, The Islamia University of Bahawalpur, Pakistan. Subsequently, all the gathered tick specimens underwent a cleansing process using 70% ethanol, followed by a final rinse with distilled water to remove any exterior contamination. Various morphological features were observed under the stereomicroscope (SZ61, Olympus Corporation, Tokyo, Japan) according to the standard identification keys (Hoogstraal and Varma, 1962; Kaiser and Hoogstraal, 1963; Dhanda and Kulkarni, 1969; Walker et al., 2005; Hosseini-Chegeni et al., 2013) to confirm the morphology of ticks. The ticks that were recognized based on their morphology were stored in 100% ethanol in the laboratory until they were ready to be processed for the extraction of genomic DNA (gDNA) and molecular analysis.

### Blood collection and microscopic smear examination

In addition to inspections of tick infestation, veterinary experts undertook additional examinations of all the cattle hosts in all four districts. However, in Kohat District, the tick-infested cattle hosts were considered as suspected or positive based only on their observable signs and symptoms associated with any common TBDs, such as fever, loss of appetite, diarrhea, fatigue, coughing, and decreased milk output, as determined by a veterinary official. Therefore, blood samples were obtained from the jugular vein of each cattle in all four districts and stored in labeled vacutainers coated with ethylenediaminetetraacetic acid (EDTA). A total of 16 blood samples were promptly taken and delivered to the above-stated laboratory for the purpose of microscopic examination of a thin blood smear using Giemsa staining. Briefly, a drop of blood was poured on a glass slide for preparation of blood smear. These smears were then fixed with methanol and stained with a Giemsa (10%) for 30 min. Finally, the smears were inspected under a microscope by following the procedure outlined by Aktas and Özübek (2015) to detect the bacteria inside the cytoplasm. The positive blood samples were further subjected to gDNA extraction using the standard method of phenol–chloroform (Sambrook et al., 1989), followed by molecular analyses.

### Molecular analyses of ticks and pathogens

The tick species of different life stages were identified based on their morphology and were then subjected to molecular analyses. Each tick was individually cleansed after preservation using 70% ethanol, followed by distilled water in order to remove any further contaminants from the tick’s body. Subsequently, each tick was dried separately on sterile filter paper. Afterward, each chosen tick species, including various life stages, was individually crushed using sterile scissors or blades to extract the gDNA. The gDNA from each morphologically identified tick species and collected blood samples was extracted using the standard method of phenol–chloroform (Sambrook et al., 1989). The extracted gDNA of tick species as well as blood specimens was quantified using NanoDrop (Nano-Q, Optizen, Daejeon, South Korea) and then stored at −20°C until further molecular analyses.

After that, each tick’s extracted gDNA sample was used for the amplification of the partial fragment of *cox1* gene using a pair of primers in conventional PCR (PCR: GE-96G, BIOER, Hangzhou, China) for their molecular confirmation. Similarly, the gDNA of each tick species and each blood specimen were used individually in PCR for the amplification of partial fragments of the bacterial *16S rDNA* gene. In addition, a set of primers targeting the 190-kDa outer membrane protein A (*ompA*) and the 120-kDa outer membrane protein B (*ompB*) genes were used to amplify the gDNA of each tick species and blood sample in order to determine the presence of Rickettsiales DNA. The primers and PCR cycling conditions of each specific gene are provided in **Table 1**. Each specific PCR was performed in a total volume of 25 µL, comprising 1 µL of each forward and reverse primer (concentration of 10 pmol/µL), 8.5 µL of PCR water, 2 µL (100 ng/µL) of gDNA, and 12.5 µL of Dream*Taq* MasterMix (2×) (Thermo Fisher Scientific, Inc., Waltham, MA, USA). The PCR water was used as a negative control, while *Hyalomma anatolicum* tick species and *R. massiliae* were used as a positive control in each PCR. After successful amplification, almost 2 µL of each amplified product was examined by Gel Documentation System (BioDoc-It TM Imaging Systems, UVP, LLC, Upland, CA, USA) on 1.5% (w/v) agarose gel to validate the amplification efficiency.

**Table 1:** List of primer pairs used in the current study for the molecular identification of different tick species as well as for the detection of bacterial pathogens in ticks and blood samples.

Target genes	Sequence	Thermal cycling conditions	Amplified region (bp)	References
*cox1*	***cox1*F**: GGAACAATATATTTAATTTTTGG	95°C for 5 min, 40× [95°C for 30 sec, 55°C for 1 min, 72°C for 1 min], 72°C for 5 min, 4°C for α	850 bp	Chitimia et al., 2010
	***cox1*R**: ATCTATCCCTACTGTAAATATATG			
*16S rDNA*	**EHR16SD**: GGTACCYACAGAAGAAGTCC	95°C 5 min, 40× [95°C 1 min, 55°C 1 min, 72°C 1 min], 72°C 7 min, 4°C for α	345 bp	Parola et al., 2000
	**EHR16SR**: TAGCACTCATCGTTTACAGC			
*ompA*	**Rr190.70p:** ATGGCGAATATTTCTCCAAAA	95°C 3 min, 35× [95°C 20 sec, 46°C 30 sec, 63°C 1 min], 72°C 7 min, 4°C for α	632 bp	Roux et al., 1996
	**Rr190.701n:** GTTCCGTTAATGGCAGCATCT			
*ompB*	**120-M59**: CCGCAGGGTTGGTAACTGC	95°C 3 min, 40× [95°C 30 sec, 50°C 30 sec, 68°C 1.5 min], 68°C 7 min, 4°C for α	862 bp	Roux and Raoult, 2000
	**120-807:** CCTTTTAGATTACCGCCTAA			

### Genotyping and phylogenetic analyses of the obtained sequences

Prior to sequencing, the amplified products (126 for ticks and 35 for bacterial pathogens) underwent purification using the GeneClean II DNA purification Kit (QBIOgene, Illkirch, France) following the manufacturer’s given methodology. Subsequently, all the purified products were sequenced bidirectionally. Subsequently, the obtained sequences underwent trimming and purification using SeqMan v. 5 (DNASTAR, Inc., Madison, WI, USA). Finally, nine consensus sequences (four for different tick spp., two for *Ehrlichia* spp., one for *Anaplasma* sp., and two for *Rickettsia* sp.) were generated after the successful purification of all the obtained sequences. All the consensus sequences were individually subjected to the Basic Local Alignment Search Tool (BLAST) (Altschul et al., 1990) at the National Center for Biotechnology Information (NCBI) to download the high-identity sequences in FASTA format to obtain the phylogenetic relationship of our obtained sequences with already submitted sequences. The downloaded sequences along with specific selected outgroups were aligned in BioEdit Sequence Alignment Editor V.7.0.5 (Raleigh, NC, USA) (Hall et al., 2011) using ClustalW multiple alignments (Thompson et al., 1994). Separate phylogenetic trees were constructed for each specific gene using the neighbor-joining method with a p-distance model/method by applying 1,000 bootstraps in Molecular Evolutionary Genetic Analysis (MEGA-X) software (Kumar et al., 2018).

## Results

### Description regarding hosts and ticks

**Figure 2:**
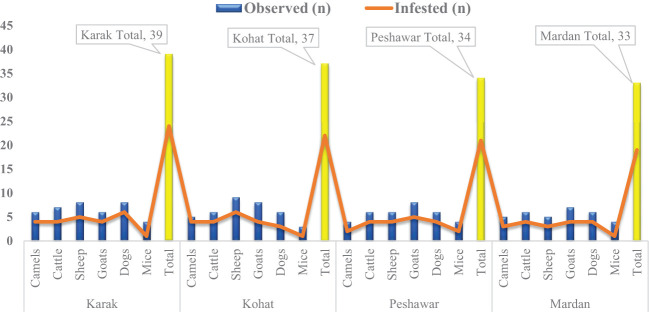
Histogram showing the details of examined and infested host animals in this study.

The tick collection was conducted on several host species, such as camels (20/143, 13.99%), cattle (25/143, 17.48%), sheep (28/143, 19.58%), goats (29/143, 20.28%), dogs (26/143, 18.18%), and mice (15/143, 10.49%) in the four demonstrated regions. However, only 86/143 (60.14%) host animals including the highest number of sheep (18/86, 20.93%) were found infested, followed by goats and dogs (17/86, 19.77%), cattle (16/86, 18.60%), and camels (13/86, 15.12%), while the least number of infested animals were mice (5/86, 5.81%) in this study. The infestation rate of the aforementioned animals was found the highest in Peshawar district (21/34, 61.76%), followed by Karak (24/39, 61.53%) and Kohat (22/37, 59.46%), while the least infestation rate of various host animals was recorded in Mardan district (19/33, 57.58%), as mentioned in **Figure 2** and **Table 2**. Overall, almost 234 partially and fully engorged ticks including the highest number of nymph stages (128, 54.70%), followed by female stage (85, 36.32%), and males (21, 8.97%) were removed from the aforementioned infested host animals. The highest number of ticks were collected in Karak district (65, 27.78%), followed by Kohat (61, 26.07%) and Peshawar (57, 24.36%), and the least were collected from Mardan district (51, 21.80%). Additionally, the highest number of ticks were removed from cattle (56, 23.93%), followed by goats (47, 20.09%), sheep (46, 19.66%), dogs (39, 16.67%), camels (38, 16.24%), and mice (8, 3.42%) (**Table 2**).

**Table 2:** Molecular detection of bacterial species in cattle’s blood and various tick species collected from different hosts in different districts of Khyber Pakhtunkhwa.

Study districts	Host(s)/vector(s)			Morphologically identified tick species	Total	Life stages of collected ticks/blood			Molecular analyses of tick species (N, F, M)/blood			Total	Screened bacteria in different tick(s) and blood		
	Type	Observed (n)	Infested (n)			Nymph (N)	Female (F)	Male (M)	N	F	M		*Ehrlichia* spp. (*16S rDNA*)	*Anaplasma* spp. (*16S rDNA*)	*Rickettsia* spp. (*ompA* and *ompB*)
Karak	Camels	6	4	*Hyalomma asiaticum*	12	7	4	1	4	3	0	7	–	–	*Candidatus Rickettsia* shennongii (4N, 3F)
	Cattle	7	4	Blood					Blood				–	–	–
				*Rhipicephalus microplus*	15	9	4	2	5	2	1	8	–	–	–
	Sheep	8	5	*Rhipicephalus turanicus*	13	6	5	2	4	3	0	7	–	–	–
	Goats	6	4	*Haemaphysalis cornupunctata*	12	7	4	1	3	2	1	6	–	–	–
	Dogs	8	6	*Rh. turanicus*	11	6	4	1	4	2	0	6	–	–	–
	Mice	4	1	*Hae. cornupunctata*	2	1	1	0	1	1	0	2	–	–	–
**Total (%)**		**39** (27.27%)	**24** (61.53%)	**65** (27.78%)		**36**	**22**	**7**	**21**	**13**	**2**	**36** (28.57%)	–	–	–
Kohat	Camels	5	4	*Hy. asiaticum*	12	6	4	2	4	2	1	7	–	–	–
	Cattle	6	4	Blood					Blood				–	*Anaplasma phagocytophilum* (4)	–
				*Rh. microplus*	14	7	5	2	4	2	1	7	–	–	–
	Sheep	9	6	*Rh. turanicus*	13	8	4	1	4	2	0	6	–	–	–
	Goats	8	4	*Hae. cornupunctata*	11	5	5	1	3	3	0	6	–	–	–
	Dogs	6	3	*Rh. turanicus*	9	5	3	1	3	2	0	5	–	–	–
	Mice	3	1	*Hae. cornupunctata*	2	1	1	0	1	0	0	1	–	–	–
**Total (%)**		**37** (25.87%)	**22** (59.46%)	**61** (26.07%)		**32**	**22**	**7**	**19**	**11**	**2**	**32** (25.40%)	–	–	–
Peshawar	Camels	4	2	*Hy. asiaticum*	5	3	2	0	2	1	0	3	–	–	–
	Cattle	6	4	Blood					Blood				–	–	–
				*Rh. microplus*	15	7	6	2	4	3	1	8	–	–	–
	Sheep	6	4	*Rh. turanicus*	12	6	5	1	3	3	0	6	–	–	–
	Goats	8	5	*Hae. cornupunctata*	13	7	5	1	3	3	1	7	–	–	–
	Dogs	6	4	*Rh. turanicus*	9	5	4	0	3	2	0	5	–	–	–
	Mice	4	2	*Hae. cornupunctata*	3	2	1	0	1	1	0	2	–	*A. phagocytophilum* (1N, 1F)	–
**Total (%)**		**34** (23.78%)	**21** (61.76%)	**57** (24.36%)		**30**	**23**	**4**	**16**	**13**	**2**	**31** (24.60%)	–	–	–
Mardan	Camels	5	3	*Hy. asiaticum*	9	5	3	1	3	2	0	5	–	–	–
	Cattle	6	4	Blood					Blood				–	–	–
				*Rh. microplus*	12	7	4	1	4	2	0	6	–	–	–
	Sheep	5	3	*Rh. turanicus*	8	5	3	0	3	1	0	4	*Ehrlichia* (3N, 1F)	–	–
	Goats	7	4	*Hae. cornupunctata*	11	5	5	1	3	2	1	6	*Ehrlichia* (3N, 2F, 1M)	–	–
	Dogs	6	4	*Rh. turanicus*	10	7	3	0	4	1	0	5	*Ehrlichia canis* (4N, 1F)	–	–
	Mice	4	1	*Hae. cornupunctata*	1	1	0	0	1	0	0	1	–	–	–
**Total (%)**		**33** (23.08%)	**19** (57.58%)	**51** (21.80%)		**30**	**18**	**3**	**18**	**8**	**1**	**27** (21.43%)	**Rickettsiales DNA in 24** (19.05%) ticks		
**Overall (%)**		Goats (29, 20.28%) Sheep (28, 19.58%) Dogs (26, 18.18%) Cattle (25, 17.48%) Camels (20, 13.99%) Mice (15, 10.49%) **143**	Sheep (18, 20.93%) Goats (17, 19.77%) Dogs (17, 19.77%) Cattle (16, 18.60%) Camels (13, 15.12%) Mice (5, 5.81%) **86** (60.14%)	*Rh. turanicus* (85, 36.32%) *Rh. microplus* (56, 23.93%) *Hae. cornupunctata* (55, 23.50%) *Hy. asiaticum* (38, 16.24%) **234**		**128** (54.70%)	**85** (36.32%)	**21** (8.97%)	**74** (58.73%)	**45** (35.71%)	**7** (5.55%)	**126** (53.85%)	*Ehrlichia* spp. in *Rh. turanicus* and *Hae. cornupunctata* tick species: 15 (10N, 4F, 1M; 11.90%)	*A. phagocytophilum* in blood of cattle (4/16, 25.00%). *A. phagocytophilum* in *Hae. cornupunctata* tick species: 2 (1N, 1F; 1.59%)	*Ca. R.* shennongii in *Hy. asiaticum* tick species: 7 (4N, 3F; 5.56%)
				Cattle (56, 23.93%) Goats (47, 20.09%) Sheep (46, 19.66%) Camels (38, 16.24%) Dogs (39, 16.67%) Mice (8, 3.42%) **234**											

### Morpho-molecular analyses of collected specimens

The 234 tick specimens were classified into three different genera based on their morphological characteristics: *Hyalomma*, *Rhipicephalus*, and *Haemaphysalis*. The largest number of ticks belonged to the specific species of *Rhipicephalus turanicus* (85, 36.32%), followed by *Rhipicephalus microplus* (56, 23.93%), *Haemaphysalis cornupunctata* (55, 23.50%), and *Hyalomma asiaticum* (38, 16.24%). Out of a total of 234 tick species, 126 (53.85%) representative tick species were molecularly confirmed using partial fragments of *cox1* gene (**Table 2**).

### Microscopic examination of blood

Intra-cytoplasmic inclusion bodies were observed in the prepared smears of almost 4/16 (25.00%) of cattle from Kohat district (**Supplementary Figure 1**); however, all the remaining blood samples of 12/16 (75.00%) were found negative by blood smear examination. Genomic DNA were extracted from all the positive blood samples.

### Phylogenetic analyses of the obtained tick sequences

All the amplified products of partial fragments of *cox1* gene from each tick species were sequenced. Since all the sequences acquired from each morphologically identifiable tick species were similar, hence a single consensus sequence was used for each tick species in their phylogenetic analysis. In conclusion, a total of four consensus and purified sequences were found for the four tick species stated before, which belong to three different genera: *Rh. turanicus*, *Rh. microplus*, *Hae. cornupunctata*, and *Hy. asiaticum*.

**Figure 3:**
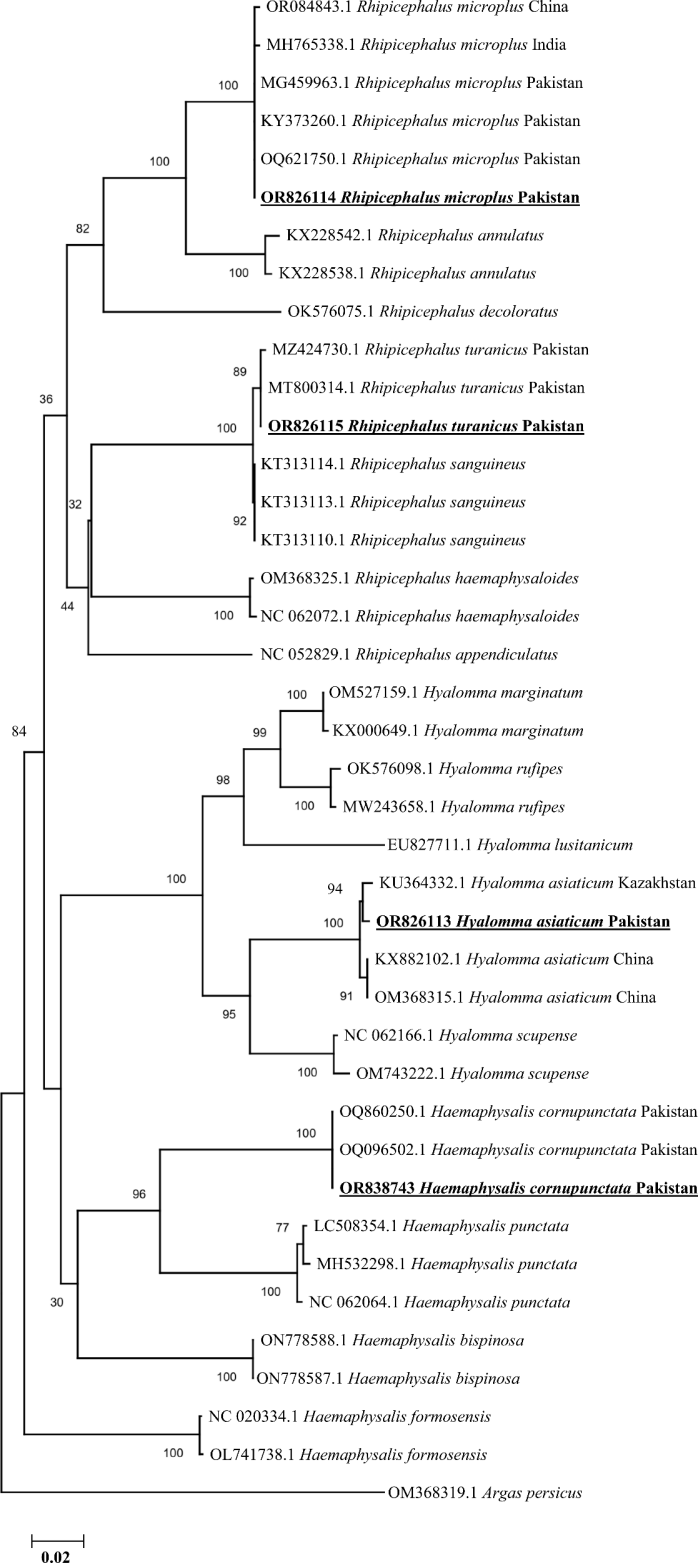
Phylogenetic analyses of tick species through the partial fragment of *cox1* gene using neighbor-joining method and p-distance model. The sequences of the obtained tick species in the current study are marked as bold and underlined fonts (OR826113, OR826114, OR826115, and OR838743). The soft tick specie, *Argus persicus*, (OM368319.1) was chosen as an outgroup.

The consensus sequence of *Rh. turanicus* (723 bp) showed 99.72%–100% identity with the corresponding species by the BLAST result and clustered in the clade of the same species reported from Pakistan in the phylogenetic tree. The BLAST result of the consensus sequence of *Rh. microplus* (761 bp) tick species had maximum identity (99.74%–100%) with *Rh. microplus* tick species reported from China, Pakistan, and India, showing a close phylogenetic relationship with *Rh. microplus* in its phylogenetic analyses. Similarly, the consensus sequence of each *Hae. cornupunctata* (633 bp) and *Hy. asiaticum* (758 bp) showed 100% and 99.21%–99.34% identities with their corresponding species, respectively, and clustered phylogenetically with their corresponding related species reported from Kazakhstan and China (**Figure 3**).

### Detection of bacterial DNA and their phylogenetic analyses

Consequently, the extracted DNA from the selected tick species and blood was tested for the detection of different bacterial pathogens including the highest existing rate of *Ehrlichia* spp. (15/126, 11.90% in ticks), followed by *Anaplasma* spp. (4/16, 25.00% in blood and 2/126, 1.59% in ticks) and *Rickettsia* spp. (7/126, 5.56% in ticks). However, no Rickettsiales DNA belonging to the genus *Rickettsia* were detected in the DNA of all the collected blood specimens.

### *Rickettsia* spp. and phylogenetic analysis

**Figure 4:**
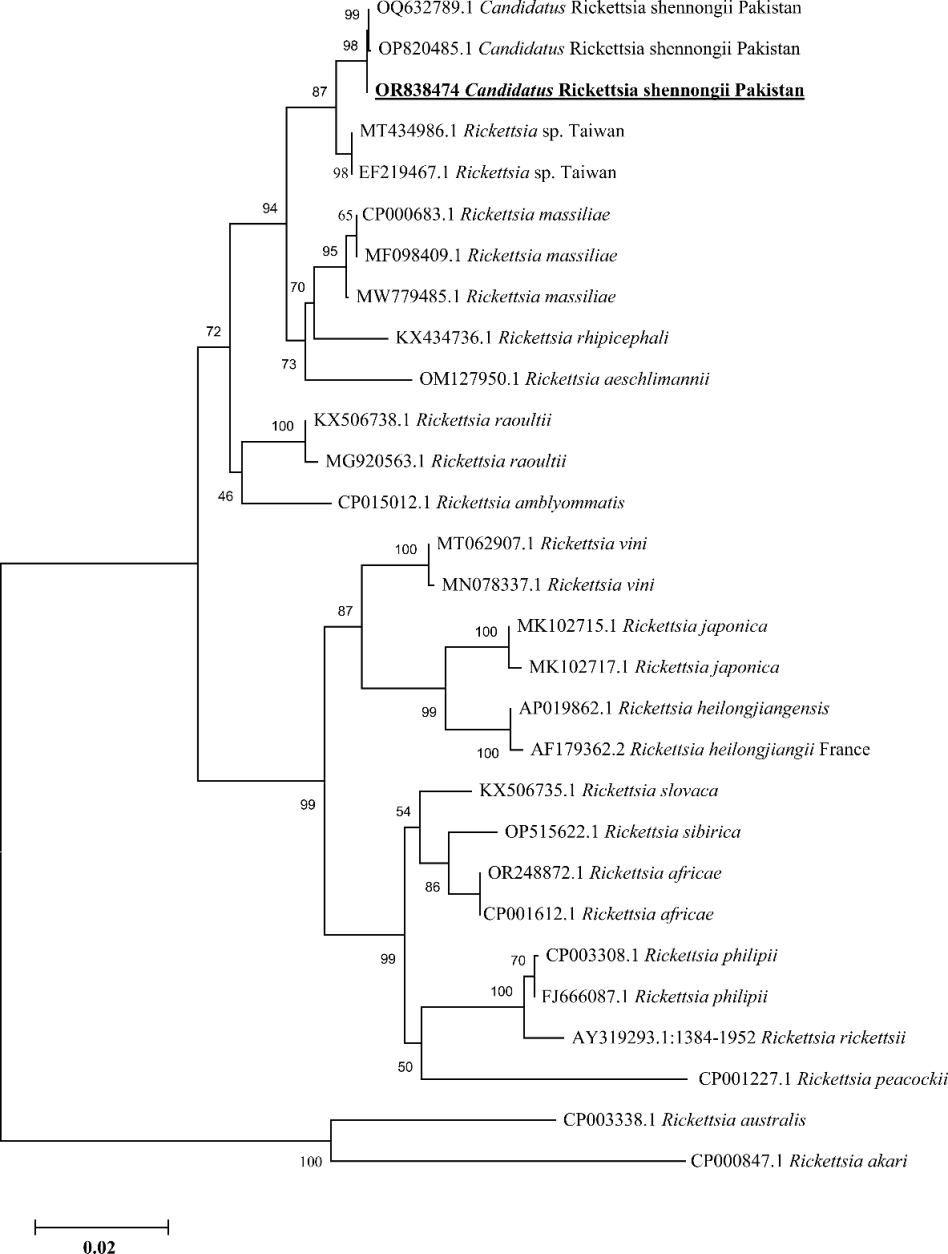
Phylogenetic analyses of *Candidatus Rickettsia* shennongii through the partial fragment of *ompA* gene using neighbor-joining method and p-distance model. The sequence of the obtained *Ca. R.* shennongii in the current study are marked as bold and underlined fonts (OR838474). *Rickettsia australis* (CP003338.1) and *Rickettsia akari* (CP000847.1) were chosen as outgroup sequences.

Based on partial fragments of *ompA* and *ompB*, the *Ca. R.* shennongii was identified in *Hy. asiaticum* tick species (7/126, 5.56%) collected from camels in Karak district, and all the amplified products of each gene (*ompA* and *ompB*) were sequenced for their phylogenetic analyses. Due to the homologous/identical nature of all the obtained sequences, a single consensus sequence of each gene (*ompA* and *ompB*) was used to retrieve highly similar sequences to obtain their phylogenies. By the BLAST results, our obtained single consensus *ompA* sequence showed 99.29%–100% identity with the *Ca. R.* shennongii and *Rickettsia* sp. reported from Pakistan and Taiwan, respectively, that clustered with the corresponding species in the phylogenetic tree (**Figure 4**).

**Figure 5:**
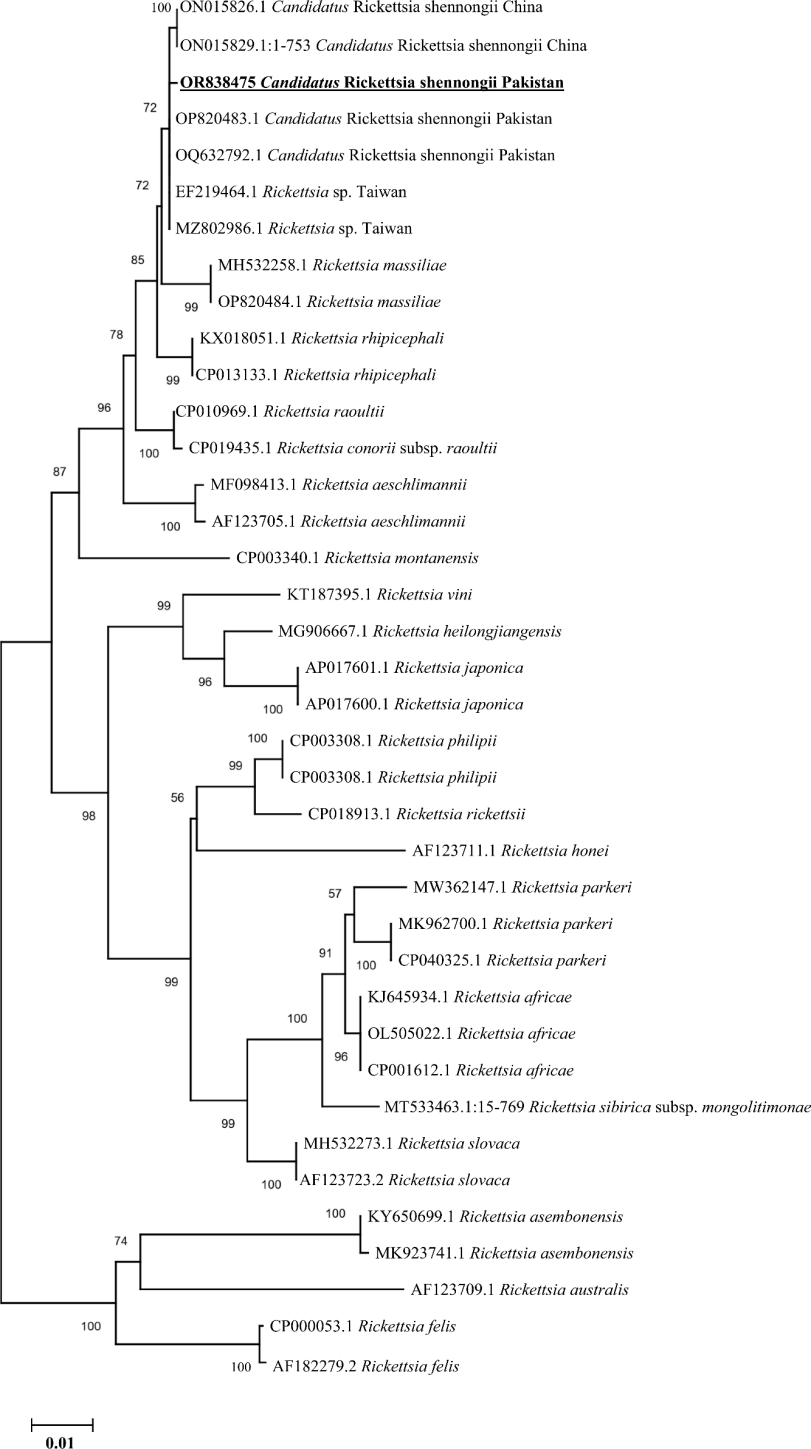
Phylogenetic analyses of *Candidatus Rickettsia* shennongii through the partial fragment of *ompB* gene using neighbor-joining method and p-distance model. The sequence of the obtained *Ca. R.* shennongii in the current study is marked as bold and underlined fonts (OR838475). *Rickettsia felis* (CP000053.1 and AF182279.2) were chosen as outgroup sequences.

Similarly, the BLAST results of the obtained single consensus *ompB* sequence showed the maximum identity in the range of 99.73%–99.87% with the *Ca. R.* shennongii and *Rickettsia* sp. that were clustered with their corresponding species reported from Pakistan, China (the mainland and Taiwan), and Sri Lanka in the phylogenetic tree (**Figure 5**).

### Phylogenetic analysis of *Anaplasma phagocytophilum* and *Ehrlichia* spp.

**Figure 6:**
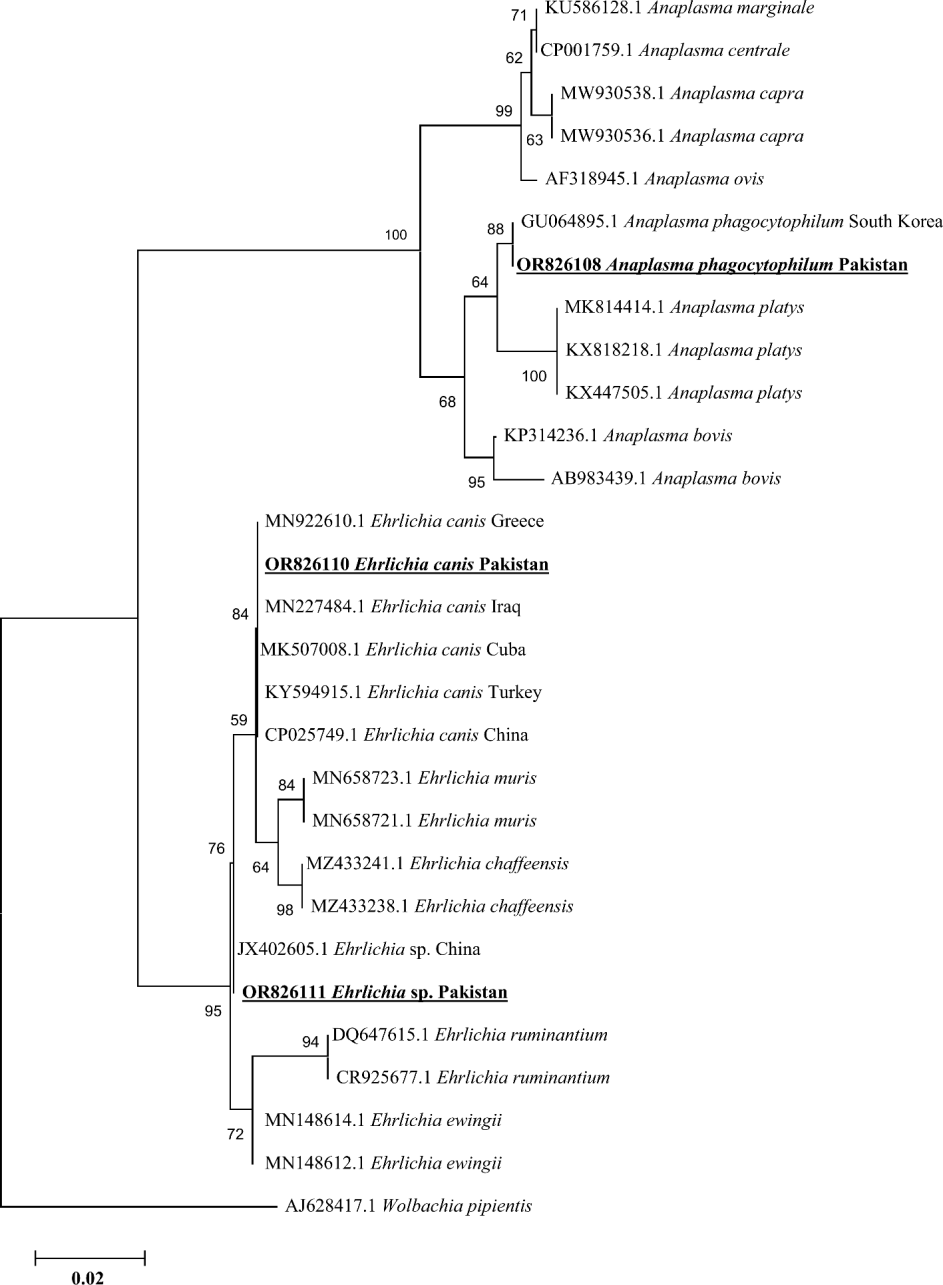
Phylogenetic analyses of *Anaplasma phagocytophilum*, *Ehrlichia canis*, and *Ehrlichia* sp. through the partial fragment of *16S rDNA* gene using neighbor-joining method and p-distance model. The sequence of the obtained *A. phagocytophilum*, *E. canis*, and *Ehrlichia* sp. in the current study are marked as bold and underlined fonts (OR826108, OR826110, and OR826111), respectively. *Wolbachia pipientis* (AJ628417.1) was chosen as an outgroup sequence.

Based on consensus sequences of *16S rDNA* partial fragments, the *A. phagocytophilum* and *Ehrlichia* spp. (*Ehrlichia canis* and *Ehrlichia* sp.) were confirmed. The obtained consensus sequence of *16S rDNA* partial fragment was obtained from the DNA of cattle’s blood and *Hae. cornupunctata* and was used to download the high-identity sequences from the NCBI using the BLAST, and our obtained consensus sequence showed a maximum of 99.67% identity with *A. phagocytophilum* reported from South Korea and clustered phylogenetically in a clade with the same corresponding species (**Figure 6**).

In addition, we obtained two distinct consensus sequences for *Ehrlichia* spp., which were then subjected to BLAST search in the NCBI to obtain the corresponding homologous sequences for their phylogenetic analysis. One consensus sequence of *Ehrlichia* spp. showed 100% identity with the *E. canis* species and clustered in the same clade with the same corresponding species reported from Greece, Cuba, Iraq, Turkey, and China. Another consensus sequence had the highest identity (99.71%) with the same unidentified species of *Ehrlichia* reported from China, which was clustered with the corresponding *Ehrlichia* sp. in the phylogenic tree (**Figure 6**).

## Discussion

In Pakistan, small-scale animal husbandry is prevalent in rural and economically impoverished communities, supporting both domestic and commercial purposes. In order to accomplish this target, diverse animals such as camels, cattle, buffaloes, sheep, and goats are imported from neighboring countries due to their better milk production capabilities. As a result, farmers have embraced commercial farming methods that use advanced technology. However, a wide range of Ixodid ticks and TBPs are prevalent in distinct ecological and geographical areas of Pakistan (Karim et al., 2017; Ghafar et al., 2020b; Atif et al., 2022), as exotic animals are core affected and vulnerable to ticks and their associated TBPs even in a controlled environment as described by Young et al. (1988), significantly impacting the farming community in Pakistan. In recent years, numerous TBPs belonging to different genera including *Rickettsia*, *Ehrlichia*, *Anaplasma*, *Coxiella*, and *Bartonella* have been documented in a variety of ticks and their animal hosts from various regions of Pakistan (Iqbal et al., 2019; Ali et al., 2023b; Khan et al., 2023; Rizwan et al., 2023; Shehla et al., 2023b), showing considerable public health interest. Their phylogenic affiliations have evolved much and are still evolving with the passage of time. Hence, this study was aimed for the molecular identification of various tick species and their Rickettsiales bacteria in camels, cattle, goats, sheep, dogs, and mice from the specified targeted area because the aforementioned host animals except mice are considered as companions of humans, which might play an important role in the natural transmission cycle and dispersal of different ticks and their associated TBPs. Herein, four tick species, namely, *Rh. turanicus*, *Rh. microplus*, *Hae. cornupunctata*, and *Hy. asiaticum*, were genetically identified successfully using *cox1* partial sequences. Additionally, the molecular identification of bacterial pathogens including *Ca. R.* shennongii (based on *ompA* and *ompB*), *E. canis* and unidentified *Ehrlichia* sp. (based on *16S rDNA*), and *A. phagocytophilum* (based on *16S rDNA*) was achieved. Moreover, this is the first-ever genetic evidence of *Hy. asiaticum* tick species carrying the *Ca. R.* shennongii from Pakistan.

The majority of the examined animals in this study were camels, although the infestation rate of ticks was found higher in sheep as compared to other host animals, which aligns with the prior research findings documented from North Africa (Khamassi-Khbou et al., 2021), and the high infection rate may be attributed to the abundance of a variety of sheep breeds and the presence of long wool on their bodies, which enables the ticks to thrive in the specific research area.

Four tick species belonging to three genera including *Rhipicephalus*, *Hyalomma*, and *Haemaphysalis* were found on the animal host bodies in the current study because the targeted study locations are comprised of arid to semi-arid regions, and their climatic conditions vary due to having four seasons (spring, winter, summer, and fall). Climate variability and global warming significantly influence the proliferation and distribution of tick species (Leger et al., 2013; Gilbert et al., 2014). The three aforementioned genus species exhibited dominance in the research region, perhaps attributed to the aggregation of various animals in overcrowded shelters/farms and the simultaneous grazing of diverse host animals in the studied areas. In the current study, the targeted region predominantly exhibited ticks belonging to the genus *Rhipicephalus*, and these findings were in accordance with previously published data from Pakistan (Obaid et al., 2023). More specifically, the *Rh. turanicus* tick species was predominant as compared to other tick species that were infesting both sheep and dogs in the targeted study locations, and these findings are parallel to the previous findings of Ceylan et al. (2021) and Alam et al. (2022).

Due to the presence of similarities and juvenile stages, it is inadequate to achieve the accurate morphological identification of tick species. Therefore, genetic markers such as mitochondrial *cox1* gene have been successfully proven as reliable for the molecular confirmation of different ticks (Filipe et al., 2021; Zhao et al., 2020). In the current study, we report the *Hy. asiaticum* as the first molecular report from Pakistan, as it was reported morphologically prior to this investigation (Afridi et al., 2023), and there was no molecular evidence from Pakistan. However, the remaining three species including *Rh. turanicus*, *Rh. microplus*, and *Hae. cornupunctata* were already morpho-molecularly reported from various regions of Pakistan. Phylogenetically, the obtained partial sequences of *cox1* gene from the aforementioned tick species revealed a close evolutionary relationship with their corresponding species reported from Kazakhstan, Pakistan, India, and China, and the accuracy regarding their topology was supported by previously published data.

The results of our investigation confirmed the presence of Rickettsiales DNA in the investigated tick species, and it is worth noting that this kind of bacterial DNA has been previously detected in other tick species globally (Yang et al., 2016; Shao et al., 2021; Hu et al., 2022; Ali et al., 2023b). More specifically, the *Ca. R.* shennongii based on *ompA* and *ompB* genes were presented for the first time from the *Hy. asiaticum* tick species collected from camels in the current study, and these findings were parallel to the previous findings that have presented the carrier nature of *Hy. asiaticum* for *Rickettsia* spp. collected from camels (Batu et al., 2020). Therefore, these considerations suggest that camels are likely reservoirs for *Rickettsia* species in the study area, although the pathogenicity of *Ca. R.* shennongii is still undetermined (Shehla et al., 2023a). Hence, extensive research work is needed to update the scientific community regarding its pathogenicity.

The *A. phagocytophilum* bacterium has a broad range of susceptible hosts and may induce illness in ruminants, horses, dogs, and cats. Moreover, it is recognized as a new human pathogen of growing significance worldwide (Stuen et al., 2002; Strle, 2004; Dumler, 2005). In the past, *Anaplasma* spp. have been genetically identified in the blood of numerous animals including cattle in different regions of the globe, using different genetic markers (Dugat et al., 2015; Bekloo et al., 2018; Byaruhanga et al., 2018; Iqbal et al., 2019; Iatta et al., 2021; Hu et al., 2022). Using the highly conserved *16S rDNA* marker, we have molecularly detected *A. phagocytophilum* in the blood of cattle. These results provide compelling evidence that cattle might serve as a reservoir for the *Anaplasma* bacterium. In addition, we molecularly identified *A. phagocytophilum* from ticks (*Hae. cornupunctata*) collected from mice in livestock farms in Peshawar district, and these results are consistent with a recently published work (Santos et al., 2009) A study indicated that mice may have significant involvement in maintaining the active cycles of this bacterial pathogen. The presence of mice in animal farms in Peshawar district may be a contributing factor in the transmission of *Anaplasma* bacteria to livestock animals from mice through the ticks, as previous research has shown the presence of *Anaplasma* species in ticks collected from cattle in the same district (Khan et al., 2022).

Sheep, goats, and dogs roam freely outdoors in both rural and urban areas of the study locations to find food and hunt. This raises the risk of tick infestation and the spread of various TBPs such as *Ehrlichia* spp. This bacterium has been found worldwide in the blood of animals and in ticks that infest host animals including cattle, sheep, goats, dogs, and horses (Dumler et al., 2001; Bekloo et al., 2018; Malik et al., 2018; Hu et al., 2022). Our findings declared the existence of *Ehrlichia* genus especially *E. canis* and unidentified *Ehrlichia* sp. in *Rh. turanicus* and *Hae. cornupunctata* ticks, collected from sheep, dogs, and goats. These findings are in accordance with the previous research works performed by Ali et al. (2023b); Malik et al. (2018), and Iatta et al. (2021) in Pakistan.

Phylogenetically, the topology of various Rickettsiales bacteria including *Ca. R.* shennongii, *A. phagocytophilum*, and *Ehrlichia* spp. (*E. canis* and *Ehrlichia* sp.) was supported by previous studies (Yang et al., 2016; Iqbal et al., 2019; Shao et al., 2021; Ali et al., 2023b; Shehla et al., 2023a). The occurrence of Rickettsiales DNA in different animals suggests that there is a wide range of both identified and unidentified bacteria in Pakistan that may be transmitted between animals and humans. Therefore, it is crucial to carry out more extensive research studies to record these pathogenic bacteria and investigate their pathogenic roles in the natural environment. Moreover, it is crucial to conduct in-depth “One-Health” research to better understand the epidemiology, transmission, and pathogenicity of Rickettsiales bacteria in the country. The One-Health approach is especially important for devising strategies to manage tick infestations and associated TBDs for the aforementioned objectives.

## Conclusion

This work presents the first genetic analysis of the *Hy. asiaticum* tick species in Pakistan using the *cox1* genetic marker. In addition, this work reveals the genetic confirmation of tick species such as *Rh. turanicus*, *Rh. microplus*, and *Hae. cornupunctata*. Rickettsiales bacteria including *Ca. R.* shennongii, *A. phagocytophilum*, *E. canis*, and *Ehrlichia* sp. are molecularly reported in the aforementioned tick species. Moreover, the *Ca. R.* shennongii was reported for the first time in *Hy. asiaticum* tick species from Pakistan. Furthermore, the *A. phagocytophilum* was also genetically isolated from the DNA of cattle’s blood. The presence of diverse tick species and detection of Rickettsiales pathogen in ticks as well as cattle’s blood might suggest a lack of long-term surveillance that highlights the need for additional comprehensive studies regarding the epidemiology and evolutionary status of various ticks and their associated TBPs in Pakistan.

## Data availability statement

The datasets presented in this study can be found in online repositories (https://www.ncbi.nlm.nih.gov/genbank/). The names of the repository/repositories and accession number(s) can be found below: *Hy. asiaticum* (OR826113), *Rh. microplus* (OR826114), *Rh. turanicus* (OR826115), *Hae. cornupunctata* (OR838743), *A. phagocytophilum* (OR826108), *Ca. R. shennongii* (*ompA*; OR838474, *ompB*; OR838475), *E. canis* (OR826110), and *Ehrlichia* sp. (OR826111).

## Ethics statement

The requirement of ethical approval was waived by taking the verbal and written informed consent/General permission letter from the Animal owner’s before the sampling of ticks and blood. Additionally, all the blood samples from cattle were collected by veterinary officials, and animal owner’s showed their full trust and satisfaction. This study was conducted in accordance with the local legislation and institutional requirements. 

## Author contributions

MKO: Conceptualization, Data curation, Formal analysis, Investigation, Methodology, Resources, Software, Validation, Writing – original draft, Writing – review & editing. ShS: Conceptualization, Data curation, Formal analysis, Investigation, Methodology, Resources, Software, Validation, Writing – original draft, Writing – review & editing. GG: Formal analysis, Investigation, Funding acquisition, Methodology, Project administration, Resources, Software, Validation, Visualization, Writing – review & editing. MR: Funding acquisition, Project administration, Resources, Software, Validation, Writing – review & editing, Data curation, Supervision. SuS: Data curation, Writing – review & editing, Formal analysis, Methodology. 
