# Supplementary material for: Genotyping of ticks: first molecular report of Hyalomma asiaticum and molecular detection of tick-borne bacteria in ticks and blood from Khyber Pakhtunkhwa, Pakistan
Source: Front Cell Infect Microbiol. 2024 Mar 12;14:1346595. doi: 10.3389/fcimb.2024.1346595 (PMC10963394; doi:10.3389/fcimb.2024.1346595)
Supplement: Supplementary file 1 [file Presentation_1.pdf]

Supplementary Material

Genotyping of ticks: with first molecular report of *Hyalomma asiaticum*, and molecular detection of tick-borne bacteria in ticks and blood from Khyber Pakhtunkhwa, Pakistan

Muhammad Kashif Obaid[1]*, Shehla Shehla[2], Guiquan Guan[1]*, Muhammad Rashid[3], Sumaira shams[2]

*** Correspondence**: **M.K.O**; kashifobaidkanz@gmail.com, **G.G**; guanguiquan@caas.cn

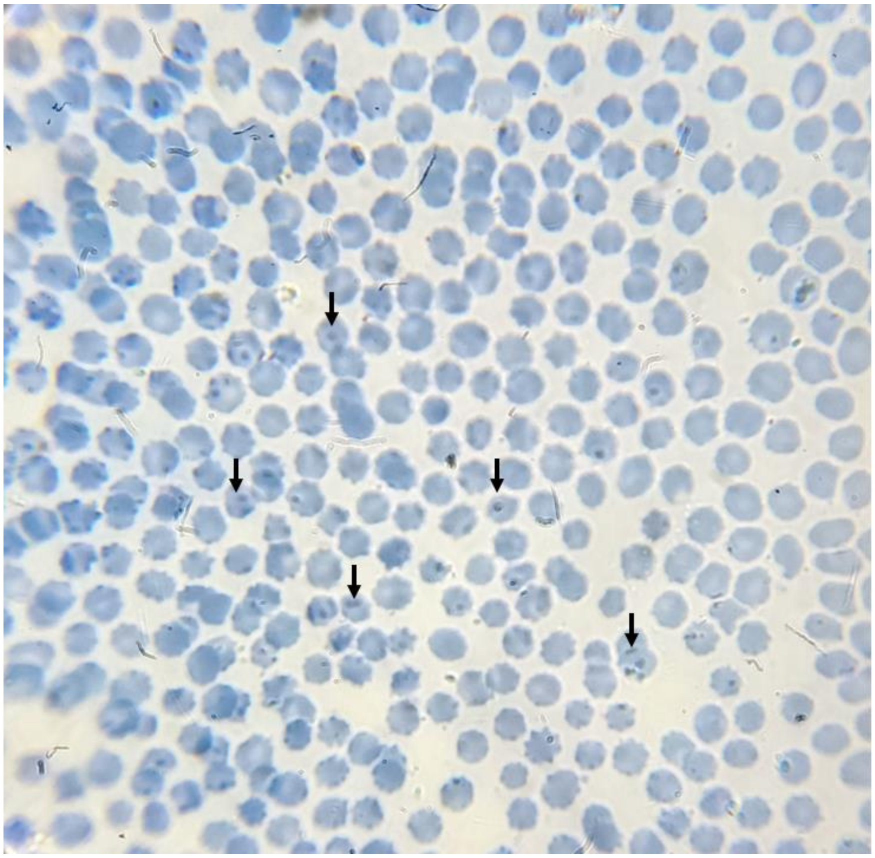

Supplementary Figure 1. Thin blood smears were stained by Giemsa (10%) for the examination of intra-cytoplasmic inclusion bodies (arrows) the in erythrocytes.
